# Brain hypoxia, neurocognitive impairment, and quality of life in people post-COVID-19

**DOI:** 10.1007/s00415-023-11767-2

**Published:** 2023-05-21

**Authors:** Damilola D. Adingupu, Ateyeh Soroush, Ayden Hansen, Rosie Twomey, Jeff F. Dunn

**Affiliations:** 1 Department of Radiology, University of Calgary, Calgary, Canada; 2 Hotchkiss Brain Institute (HBI), University of Calgary, Calgary, Canada; 3 Department Of Clinical Neurosciences, University of Calgary, Calgary, Canada; 4 Faculty of Kinesiology, University of Calgary, Calgary, Canada

**Keywords:** Brain hypoxia, Cerebral hypoxia, Post-COVID-19 condition, Cerebral tissue Oxygen saturation, Frequency-domain near-infrared spectroscopy

## Abstract

**Objective:**

Systemic hypoxia occurs in COVID-19 infection; however, it is unknown if cerebral hypoxia occurs in convalescent individuals. We have evidence from other conditions associated with central nervous system inflammation that hypoxia may occur in the brain. If so, hypoxia could reduce the quality of life and brain function. This study was undertaken to assess if brain hypoxia occurs in individuals after recovery from acute COVID-19 infection and if this hypoxia is associated with neurocognitive impairment and reduced quality of life.

**Methods:**

Using frequency-domain near-infrared spectroscopy (fdNIRS), we measured cerebral tissue oxygen saturation (S_*t*_O_2_) (a measure of hypoxia) in participants who had contracted COVID-19 at least 8 weeks prior to the study visit and healthy controls. We also conducted neuropsychological assessments and health-related quality of life assessments, fatigue, and depression.

**Results:**

Fifty-six percent of the post-COVID-19 participants self-reported having persistent symptoms (from a list of 18), with the most reported symptom being fatigue and brain fog. There was a gradation in the decrease of oxyhemoglobin between controls, and normoxic and hypoxic post-COVID-19 groups (31.7 ± 8.3 μM, 27.8 ± 7.0 μM and 21.1 ± 7.2 μM, respectively, *p* = 0.028, *p* = 0.005, and *p* = 0.081). We detected that 24% of convalescent individuals’ post-COVID-19 infection had reduced S_*t*_O_2_ in the brain and that this relates to reduced neurological function and quality of life.

**Interpretation:**

We believe that the hypoxia reported here will have health consequences for these individuals, and this is reflected in the correlation of hypoxia with greater symptomology. With the fdNIRS technology, combined with neuropsychological assessment, we may be able to identify individuals at risk of hypoxia-related symptomology and target individuals that are likely to respond to treatments aimed at improving cerebral oxygenation.

## Introduction

Coronavirus disease 2019 (COVID-19) is an acute viral illness caused by the severe acute respiratory syndrome coronavirus 2 (SARS-CoV-2). Initially, it was thought to largely impact the respiratory system. It is now recognized that COVID-19 can severely impact other organ systems, including the brain, heart, kidneys, liver, skeletal muscle, and skin [1, 2]. About 34% of people receive a neurological or psychiatric diagnosis within 6 months of COVID-19 infection [3]. Persistent symptoms after the apparent elimination of the SARS-CoV-2 have been reported [4–6]. This is termed long COVID, long-haul COVID, or post-acute COVID-19 syndrome (PACS), where after recovery from the acute phase, the individual still feels symptoms [7]. One in five people aged 18–34 years with no chronic medical conditions reported that they have not returned to their baseline health post-acute COVID-19 [5]. Persistent neurological symptoms after acute COVID-19 have also been reported in individuals who had mild disease, the majority who were never hospitalized during their acute COVID-19 illness, was healthy and active prior to infection, and are less than 50 years old [8]. Furthermore, in patients with comorbidities who had recovered from acute COVID-19, 87% reported persistent symptoms over 60 days post-recovery from acute illness [6].

It is well known that systemic inflammation can induce neuroinflammation and cellular changes, which can impair cognitive function [9, 10], and cognitive impairment has been reported in COVID-19 patients [11]. Additionally, neurological complications in COVID-19 survivors are widely reported, including mild confusion, myalgias, headaches, encephalopathy, dizziness, and loss or changes in taste and smell [3, 12]. It has been known since early in the pandemic that systemic hypoxia is a key feature of COVID-19 infection [13].

However, it is unknown if there is hypoxia in the brain, and if there is, whether this occurs with normal levels of systemic blood oxygenation. We have previously proposed that inflammatory responses within the brain can result in hypoxia and that this hypoxia can worsen inflammation, thereby creating a hypoxia-inflammation cycle [14]. We have also detected hypoxia in people with multiple sclerosis and primary biliary cholangitis, both conditions appearing to cause inflammation in the brain [15, 16].

We aimed to determine if brain hypoxia exists in individuals post-COVID-19 and if there were associations with neurocognitive impairment and quality of life. We can detect this hypoxia with a measure of cerebral tissue oxygen saturation (S_*t*_O_2_) using frequency-domain NIRS (fdNIRS) [15]. This method also provides a measure of light scattering which may relate to changes in mitochondria [17]. In a study investigating NIRS parameters in individuals with acute mountain sickness, there was an increase in light scattering without changes in absorption, and this was indicative of hypoxia-induced cerebral edema [18]. Therefore, our measure of hypoxia may also be related to vasogenic, cellular, osmotic, or interstitial brain edema. We hypothesized that a proportion of convalescent individuals post-COVID-19 will have cortical hypoxia, which will be associated with increased symptomology, and it will occur even with normal arterial oxygen saturation (S_*a*_O_2_).

We report that there was brain hypoxia in approximately 24% of individuals who were at least 8 weeks post-COVID-19 infection, despite normal arterial saturation and no signs of fever. Moreover, we show hypoxia was associated with poor neuropsychological assessment, depression, fatigue, and reduced health-related quality of life.

## Materials and methods

### Subjects

**Fig. 1:**
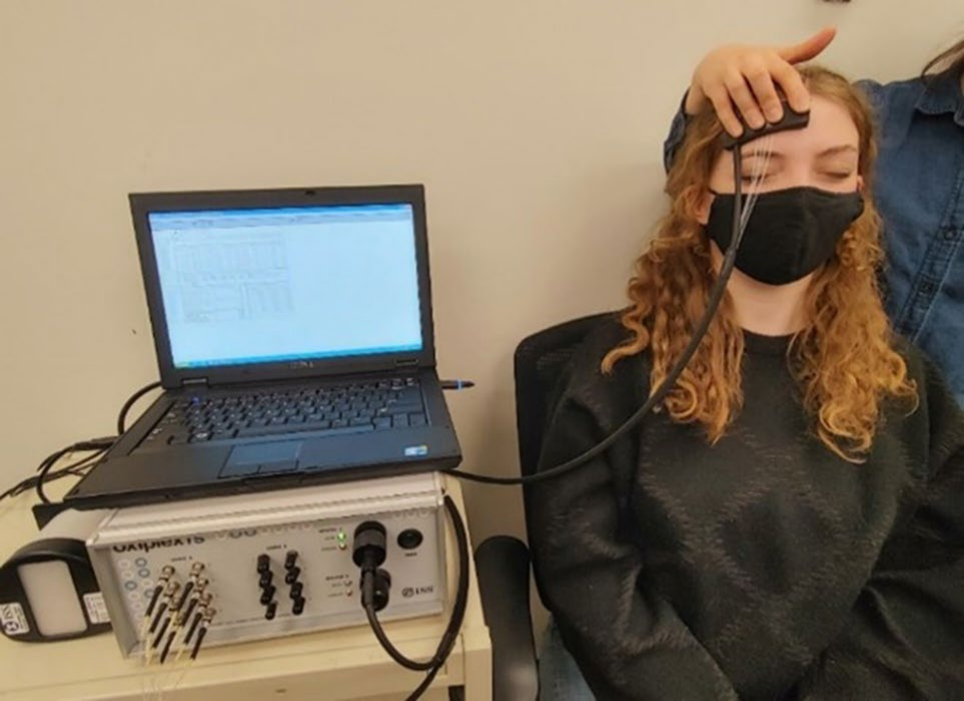
Frequency-domain near-infrared spectroscopy (ISS OxiplexTS, model 96,208, ISS Inc., Champaign, IL) used in the measurement of frontal cortical microvascular oxygenation (S_*t*_O_2_)

Healthy controls aged 18–65 years (*n* = 17) who were non-smokers (nicotine or marijuana), with no recent systemic infection, and no history of cardiovascular/vascular disease or neuropsychological disease were recruited. We recruited 34 participants who had contracted COVID-19 at least 8 weeks prior to the study visit from the general population. Exclusion criteria included smokers (nicotine or marijuana), history of cardiovascular/vascular disease, and other systemic inflammatory diseases such as inflammatory bowel syndrome, asthma, autoimmune diseases, celiac disease, glomerulonephritis, and hepatitis. Post-COVID-19 participants were screened for lingering symptoms. Participant demographics are summarized in Table 1. All participants provided written informed consent prior to the commencement of their participation. Informed consent was obtained from participants in Fig. 1 for the publication of identifying images in an online open-access publication.

**Table 1:** Demographic, oxygen saturation, light scattering and absorbance parameters, and neurocognitive measures in healthy controls and post-COVID-19 participants (mean ± SD)

	Healthy control (mean ± SD) *n* = 17	Post-COVID-19 participants (mean ± SD) *n* = 34	*p* value
Age (years)	36.2 ± 13.4	40.4 ± 13.4	0.309
Sex (% female)	53	77	
S_*a*_O_2_(%)	97.2 ± 1.2	96.7 ± 1.4	0.214
HR (BPM)	67.5 ± 8.0	71.5 ± 11.7	0.163
Tympanic temp (^o^C)	36.8 ± 0.3	37.0 ± 0.4	0.206
TOMM (/50)	49.4 ± 0.7	49.3 ± 0.8	0.629
S_*t*_O_2_ (%)	63.1 ± 3.4	60.1 ± 6.9	0.037
THb (uM)	49.8 ± 11.1	43.4 ± 10.2	0.053
HbO (uM)	31.8 ± 8.3	26.2 ± 7.5	0.028
HHb (uM)	18.1 ± 3.2	17.1 ± 4.9	0.407
*µ*_*s*_ at 690 (cm^−1^)	10.8 ± 1.4	9.7 ± 1.5	0.015
*µ*_*s*_ at 824 (cm^−1^)	9.2 ± 1.2	8.3 ± 1.3	0.018
*µ*_*a*_ at 690 (cm^−1^)	0.12 ± 0.02	0.11 ± 0.03	0.173
*µ*_*a*_ at 824 (cm^−1^)	0.12 ± 0.02	0.11 ± 0.02	0.049
SDMT-oral *z*-score	0.98 ± 1.08	− 0.62 ± 1.20	< 0.001
COWAT-FAS *z*-score	− 0.02 ± 0.62	− 0.61 ± 0.90	0.010
COWAT-Animals *z*-score	0.29 ± 0.51	− 0.26 ± 1.02	0.016
PASAT *z*-score	0.33 ± 0.68	− 0.42 ± 0.84	0.002

*HR* heart rate, *HbO* oxyhemoglobin, *HHb* deoxyhemoglobin, *THb* total hemoglobin, *PASAT* paced auditory serial addition test, *SDMT* symbol digit modality test, *StO*_*2*_ microvascular tissue oxyhemoglobin saturation, *TOMM* test of memory malingering, *µ*_*s*_ scattering coefficient, *µ*_*a*_ absorption coefficient

Data collection was initiated no sooner than 20 min after participants entered the laboratory. This time was used to obtain consent. This calm 20-min period will help minimize physiological changes that may occur from previous activities. We also asked about physical activities over the previous 6 h.

### NIRS measurement

fdNIRS measurements were taken on the frontal cortex using a quantification system called the ISS (OxiplexTS Frequency Domain Near-Infrared Spectrometer model 96,208, ISS Inc., Champaign, IL USA) (Fig. 1). The principle behind this commercially available equipment and its application is described in detail elsewhere [18–20].

Briefly, the fdNIRS probe consists of one fiber-optic detector and eight fiber-optic sources, with a source to detector separation of 2.0–3.5 cm. Source fibers emitted NIR light at 690 and 824 nm. Emitted light had an amplitude modulation frequency of 110 MHz, and light was emitted by one source at a time according to a continuous cycle wherein the eight sources alternated between being switched on and off. The estimation of the tissue absorption coefficients at multiple wavelengths enables the oxy- and deoxy-hemoglobin (HbO, and HHb) concentration to be calculated using the Beer–Lambert law. The microvascular cortical oxygenation (S_*t*_O_2_) is calculated using the formula:

1 $${\text{S}}_{t} {\text{O}}_{{2}} = \left[ {{\text{HbO}}} \right]/\left( {\left[ {{\text{HbO}}} \right] + \left[ {{\text{HHb}}} \right]} \right).$$

Prior to data collection, the fdNIRS system was warmed up for at least 30 min and the system was calibrated using a phantom calibration block with known absorption and scattering coefficients. During data acquisition, participants were asked to sit quietly and upright in a chair. The probe was placed symmetrically on both the right and left side of the participants’ forehead and data were collected for about 1 min on each side and averaged (Fig. 1). Data were collected at a rate of 2 Hz, giving a total of 120 data points per subject. The fdNIRS quantifies the absolute values for HbO and HHb. This enables microvascular tissue oxyhemoglobin saturation (S_*t*_O_2_) to be calculated, which serves as an indicator of the oxygenation status of the brain. The absolute level of absorption and scattering coefficients (*µ*_*a*_ and *µ*_*s*_, respectively) at 690 and 824 nm were determined from the measured intensity (AC or DC) and phase shift by the ISS using the theory of photon migration [21]. Details of the mathematical equation and assumptions are discussed by Hammer et al. [21].

Systemic oxygen saturation and heart rate were measured in the finger using a pulse oximetry device (Nonin Medical, Inc. Minneapolis, MN USA Model 9500 Oximeter).

Tympanic temperature measurement was taken using a tympanic thermometer (Braun Thermoscan IRT 6520 ExacTemp).

### Neuropsychological assessments

A Neuropsychological test battery was conducted on all participants. This included a quality control using a test of memory malingering (TOMM), symbol digit modality test (SDMT) oral to test visual information processing speed, control of word association test (COWAT) to test language and verbal fluency and paced auditory serial addition test (PASAT) to test attention, concentration, auditory information processing speed, and working memory.

For the COWAT, participants were asked to list as many words as they could in 1 min that began with the letters of the alphabet F, A, and then S, except for proper names or words with different endings. The score for each trial was the sum of correct responses, excluding repeats and rule breaks. The primary outcome measure for the COWAT was the sum of the correct responses for the FAS trial. This score was converted into a *z*-score using normative data from a healthy control population [22] to account for age and level of education-related effects. The calculation for z-score is demonstrated by the equation:

2 $$z\text{-score}= \frac{(\text{participants score}-(\text{predicted score}+\text{adjustment for education}))}{\text{standard deviation}}.$$

In a fourth trial for COWAT, participants were asked to list as many animals as they could that began with any letter of the alphabet. The total score was converted to a z-score using normative data with no correction for education, and *z*-score calculated by the equation:

3 $$z\text{-score}= \frac{(\text{participants score}-\text{predicted score})}{\text{standard deviation}}.$$

Equation 3 was also used for *z*-scores calculation for SDMT and PASAT.

The SDMT is a timed 90-s test, where participants used a reference key to match numbers (1–9) with nine randomized geometric shapes [23]. The total number of correctly recorded matched pairs was tallied to give an overall score, which was converted into a z-score.

For the PASAT, a recorded series of 61 numbers (1–9) was played aloud at the rate of one number every 3 s. The participants were asked to add each spoken number to the number that was presented previously. Prior to the testing trial, participants completed up to three practice trials that consisted of only 11 numbers. Participants only proceeded to the test once they demonstrated a sufficient understanding of the task. The score for the PASAT was the sum of correct responses, with a maximum score of 60 [24]. This was converted into a z-score using normative data from a healthy control population (retrieved from the PASAT manual) to account for the level of education-related effects.

### Health-related quality of life assessment, fatigue, and depression measured in post-COVID-19 participants only

Questionnaires included the health-related quality of life (HRQoL) assessment using a 36-item instrument for adults, the RAND 36-Item Short-Form Health Survey (SF-36) [25], Functional Assessment of Chronic Illness Therapy-Fatigue Scale (FACIT-F) to assess fatigue [26] and Beck Depression Inventory second edition (BDI-II).

In COVID-19 participants, HRQoL was measured using the 36-Item Short Form Survey (SF-36). Participants were asked to score their quality of life compared to what it was prior to contracting the COVID-19 infection and at the time of the visit. Anxiety and depression were measured using the Beck Depression Inventory (BDI-II) [27].

The SF-36 measures eight health concepts (physical functioning, role limitations due to physical health problems, role limitations due to personal or emotional problems, energy/fatigue, emotional wellbeing, social functioning, bodily pain, and general health perceptions) using multi-questions, 35 in total. It also includes a single question that provides an indication of perceived change in health. Participant’s response to each question is recoded so that each is scored from 0 to 100%, with higher scores indicating a more favorable health state. This questionnaire is a generic HRQoL tool that is useful for comparing general and specific populations and the relative burden of a health condition, in this case COVID-19 infection [28].

The FACIT-F (version 4) is a 13-item self-report questionnaire that measures the severity and impact of an individual’s level of fatigue during their usual daily activities over the past week. The level of fatigue is measured on a four-point Likert scale (4 = not at all fatigued to 0 = very much fatigued) [29]. The subscale scores are calculated by first reversing negatively stated items (subtracting the response from ‘4’) and then summing the raw (0–4) scores. A total score is then derived by summing subscale scores. Participants’ fatigue subscale score ranges from 0 to 52, where a lower score indicates more severe fatigue and a cut point suggesting clinically relevant fatigue set at < 34 [30, 31]. Although there is no gold standard for the measurement of fatigue, FACIT-F has been applied in conditions like cancer, HIV, lupus, rheumatoid arthritis, psoriatic arthritis, anemia, COPD, Parkinson's disease, and post-stroke [32–38], and has been shown to be valid and reliable [36, 39, 40]. We do not claim to validate the use of the FACIT-F (version 4) to “diagnose” fatigue in individuals with post-COVID-19 condition; however we use a score of < 34 as a crude indication of clinically relevant fatigue.

The BDI-II is a 21-item self-report questionnaire that assesses the extent of common depressive symptoms occurring throughout the past 2 weeks. This questionnaire uses a scale, from 0 to 3, and responses from all items are summed to give a total score from 0 to 63, with a higher score indicating greater levels of depression [27].

## Results

We recruited 17 healthy controls and 34 individuals who have had COVID-19 and were at least 8 weeks post-diagnosis of SARS-CoV-2 infection (Table 1). We had subjects sit for 20 min to help standardize for exercise. In addition, we recorded exercise over the last 24 h. Four post-COVID-19 participants in the normoxic group and two in the hypoxic group reported a period of exercise prior to the study visit. None of these six participants’ values was outside two standard deviations from their respective means. Thus, we suggest previous activity did not impact our results.

Of the 34 individuals who have had COVID-19, 19 self-reported as having persistent symptoms, defined as having at least two symptoms that suggest long COVID. The most commonly reported symptoms were fatigue and brain fog. There were no differences between healthy controls and post-COVID-19 participants for age, S_*a*_O_2_% (% arterial blood oxygen saturation), heart rate (HR) (BPM), and tympanic temperature (°C) (Table 1).

### Comparison between controls and all post-COVID-19 participants

**Fig. 2:**
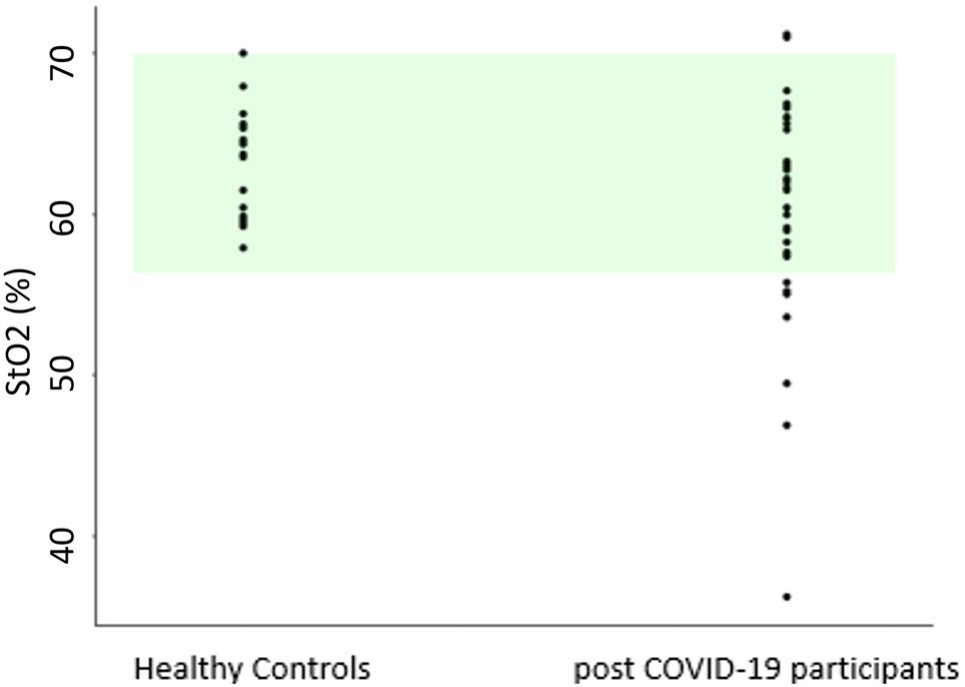
fdNIRS measurement of cortical microvascular oxygenation (S_*t*_O_2_) in healthy controls and all post-COVID-19 participants, showing the data distribution. Each dot represents one participant. Green shaded area represents 2 ± SD around the control mean. All points below the shaded area are 2 × SD below the controls (classed as hypoxic)

We compared all people post-COVID-19 with the controls (Fig. 2 and Table 1).

There are different ways of defining hypoxia. A starting point is to test whether the post-COVID-19 population has lower S_*t*_O_2_ than controls. Using the Welch’s *t* test, we show that the post-COVID-19 population is significantly different from healthy controls (lower, *p* = 0.037). The mean values between groups are 63.1 ± 3.4% and 60.1 ± 6.9% (mean ± SD) for the controls and COVID-19 groups, respectively. The respective coefficients of variation are 5.4% and 11.5%. We note that the coefficient of variation is higher in the post-COVID-19 group. If we take a conservative view that hypoxia is defined as 2xSD below the control mean, the overall mean ± SD of controls is 63.1 ± 3.4%, then anything below 56.3% would be hypoxic. There were eight post-COVID-19 participants (of 34 or 24%) who were hypoxic, while none of the controls could be classified as hypoxic.

Although nearly missing the criterion for statistical significance (*p* = 0.053), total hemoglobin in post-COVID-19 participants was lower compared with healthy controls (43.4 ± 10.2 μM vs. 49.8 ± 11.1 μM, mean ± S.D). The scattering coefficient (*µ*_*s*_) at 690 and 824 nm and the absorption coefficient (*µ*_*a*_) at 824 nm were significantly lower in post-COVID-19 participants compared with healthy controls, whereas *µ*_*a*_ at 690 nm these were not significantly different between groups.

Neuropsychological assessments all show significant impairment in the post-COVID-19 participants compared with healthy controls: symbol digit modality test (SDMT) oral (− 0.62 ± 1.20 vs. 0.98 ± 1.08 *p* < 0.001); control of word association (COWAT) for FAS (− 0.61 ± 0.90 vs. − 0.02 ± 0.62 *p* = 0.010); and animals (− 0.26 ± 1.02 vs. 0.29 ± 0.51 *p* = 0.016) and paced auditory serial addition test (PASAT) (− 0.42 ± 0.84 vs. 0.33 ± 0.68 *p* = 0.002).

### Comparison between controls and post-COVID-19 participants grouped as normoxic or hypoxic

We divided the post-COVID-19 participants into hypoxic or normoxic groups (Table 2). A one-way ANOVA, when data was normally distributed, or the Kruskal–Wallis test was carried out between healthy control, and normoxic and hypoxic groups. Hypoxic participants were measured on average 7 months (range 3–15) after infection, and normoxic participants 8 months (range 2–19) after infection. There were significant differences between normoxic and hypoxic post-COVID-19 groups for fdNIRS parameters S_*t*_O_2_ (*p* < 0.001), oxyhemoglobin (*p* = 0.007), and *µ*_*s*_ at 690 and 824 nm (*p* = 0.020 and *p* = 0.031, respectively). In post hoc analysis, there was no significant difference in S_*t*_O_2_ between normoxic post-COVID-19 participants and healthy controls; however, as expected, hypoxic post-COVID-19 participants had lower S_*t*_O_2_ compared with healthy controls and normoxic post-COVID-19 participants. HbO was significantly lower in hypoxic post-COVID-19 participants compared with healthy controls, and there were no significant differences between normoxic post-COVID-19 participants vs. healthy controls and normoxic vs. hypoxic post-COVID-19 participants (Table 2).

**Table 2:** Healthy controls compared with post-COVID-19 participants sub-divided into normoxic and hypoxic groups

	Healthy control (mean ± SD) (*n* = 17)	Normoxic post-COVID-19 (mean ± SD) (*n* = 26)	Hypoxic post-COVID-19 (mean ± SD) (*n* = 8)	Healthy control vs. normoxic post-COVID-19 *p* value	Healthy control vs. hypoxic post-COVID-19 *p* value	Normoxic post-COVID-19 vs. Hypoxic post-COVID-19 *p* value
Age (years)	36.2 ± 13.4	37.5 ± 13.0	49.6 ± 10.6	N/A	N/A	N/A
S_*a*_O_2_ (%)	97.2 ± 1.2	96.7 ± 1.4	96.6 ± 1.4	N/A	N/A	N/A
HR (BPM)	67.5 ± 8.0	71.8 ± 11.9	70.5 ± 12.1	N/A	N/A	N/A
Tympanic temp (°C)	36.8 ± 0.3	36.9 ± 0.4	36.9 ± 0.3	N/A	N/A	N/A
TOMM (/50)	49.4 ± 0.7	49.3 ± 0.8	49.4 ± 0.8	N/A	N/A	N/A
S_*t*_O_2_ (%)	63.1 ± 3.4	62.8 ± 4.0	50.9 ± 6.7	> 0.999	< 0.001	< 0.001
THb (uM)	49.8 ± 11.1	43.9 ± 9.2	41.6 ± 13.6	N/A	N/A	N/A
HbO (uM)	31.7 ± 8.3	27.8 ± 7.0	21.1 ± 7.2	0.028	0.005	0.081
HHb (uM)	18.1 ± 3.2	16.1 ± 2.9	20.5 ± 8.2	N/A	N/A	N/A
*µ*_*s*_ at 690 (cm^−1^)	10.8 ± 1.4	9.5 ± 1.5	10.4 ± 1.2	0.018	> 0.999	0.550
*µ*_*s*_ at 824 (cm^−1^)	9.2 ± 1.2	8.2 ± 1.3	8.7 ± 1.0	0.025	0.795	> 0.999
*µ*_*a*_ at 690 (cm^−1^)	0.12 ± 0.02	0.11 ± 0.02	0.12 ± 0.04	N/A	N/A	N/A
*µ*_*a*_ at 824 (cm^−1^)	0.12 ± 0.02	0.11 ± 0.02	0.11 ± 0.03	N/A	N/A	N/A
SDMT-oral *z*-score	0.98 ± 1.08	− 0.69 ± 1.30	− 0.36 ± 0.79	< 0.001	0.037	0.790
COWAT-FAS *z*-score	− 0.02 ± 0.62	− 0.64 ± 0.93	− 0.49 ± 0.81	N/A	N/A	N/A
COWAT-animals *z*-score	0.29 ± 0.51	− 0.37 ± 1.05	0.18 ± 0.83	0.050	0.963	0.302
PASAT *z*-score	0.33 ± 0.68	− 0.33 ± 0.85	− 0.76 ± 0.75	0.029	0.010	0.415

Significant (*p* ≤ 0.05) post hoc analysis was carried out where there were significant differences between groups (one-way ANOVA *p* ≤ 0.05). N/A indicates the ANOVA/Kruskal–Wallis was not significant. Tukey HSD test was used where parameters were normally distributed, otherwise Dunn test was computed

N/A: not applicable, as the one-way ANOVA or the Kruskal–Wallis test carried out showed no significant differences between healthy control, and normoxic and hypoxic groups

It may be that we should use age as a covariate. Although nearly missing the criterion for statistical significance (*p* = 0.054), hypoxic post-COVID-19 participants had a higher age, compared with the normoxic group and healthy controls (Table 2). When we did a univariate analysis with S_*t*_O_2_ as the dependent value, group as the fixed factor and age as a cofactor, group was close to being significantly different (*p* = 0.052), while age was significant (*p* < 0.001).

When age is defined by groups of 10 years (e.g., 20–29, 30–40, 40–50, and 50–63 years), there are eigjt independent groups (4 controls and 4 post-COVID-19 groups). A univariate general linear model (GLM) with Bonferroni post hoc tests indicated that S_*t*_O_2_ for the control age group 20–30 years (group 1) was higher than those of post-COVID-19 age groups 40–50 and 50–63 years, and that of the post-COVID-19 group 20–30 years was higher than those of post-COVID-19 age groups 40–50 and 50–63 years. If we group by age, we can identify hypoxic individuals by calculating how many are 2xSD below the control mean. The S_*t*_O_2_ threshold for hypoxia in the age groups are as follows: 20–30 (57.7%), 30–40 (55.1%), 40–50 (56.0%) and 50–63 years (57.1%). In these four age groups, the number of COVID-19 subjects that were hypoxic was 1, 1, 4, and 3, respectively, or 9 in total (26%). There were no controls that would be classified as being hypoxic.

In summary, if we do a simple comparison of means, the controls, and post-COVID-19 groups S_*t*_O_2_ values were different and very close to being different with a univariate analysis of variance with age as a cofactor. A clearer picture emerges if we look at how many individuals in the different groups are defined as hypoxic by being greater than 2xSD below the control mean. When we adjust for age or not, the number is 26% or 24%, respectively. These data indicate that approximately ¼ of people post-COVID-19 have significant hypoxia in the brain. Given these results, we will use the conservative cutoff for hypoxia (56.3%) for all further analysis.

Scattering coefficients at 690 and 824 nm were significantly lower in normoxic post-COVID-19 participants compared with healthy controls; however, post hoc analysis did not show a difference between hypoxic post-COVID-19 participants compared with healthy controls. There was a trend for the hypoxic post-COVID-19 participants to be older than healthy controls and normoxic post-COVID-19 participants.

There was no significant difference in S_*a*_O_2_ between the hypoxic and normoxic post-COVID-19 groups (*p* = 0.392) *p* = Table 2). HR, tympanic temperature, total hemoglobin (THb), and deoxyhemoglobin (HHb) were not different between groups.

There were significant differences between groups for neurocognitive measures SDMT-oral (*p* < 0.001), COWAT-Animals (*p* = 0.047), and PASAT (*p* = 0.006). SDMT-oral and PASAT z-scores were significantly lower in both normoxic and hypoxic post-COVID-19 participants compared with healthy controls (Table 2). The COWAT-Animals z-score was significantly lower in normoxic post-COVID-19 participants compared with healthy controls, and there was no detectable difference between hypoxic post-COVID-19 participants and healthy controls. There was a trend for COWAT-FAS *z*-score to be different between groups.

Health-related quality of life measured only in post-COVID-19 participants was significantly lower across multiple domains in the hypoxic vs. normoxic group (Table 3) and these differences were clinically meaningful. Physical functioning, role limitations due to physical health problems, social functioning, and general health were substantially lower in the hypoxic group. Fatigue measured using the FACIT-F was particularly severe in the hypoxic group (Table 3, where a score of < 34 is considered clinically significant, and these individuals scored 12 ± 9). There was no difference in depression scores between groups. The reported numbers of persistent COVID-19 symptoms did not differ between hypoxic and normoxic post-COVID-19 participants (5 ± 5 vs. 7 ± 5).

**Table 3:** Health-related quality of life (HRQoL) assessment using a 36-item instrument for adults, the RAND 36-Item Short-Form Health Survey (SF-36), the Functional Assessment of Chronic Illness Therapy-Fatigue Scale (FACIT-F), and Beck Depression Inventory second edition (BDI-II) measured in post-Covid participants sub-divided into hypoxic and normoxic

	Normoxic post-COVID-19 participants (mean ± SD) *n* = 26	Hypoxic post-COVID-19 participants (mean ± SD) *n* = 8	*p* value
Physical functioning	69 ± 33	31 ± 20	0.003
Role limitation-physical	54 ± 46	0 ± 0	< 0.001
Role limitation-emotional	60 ± 42	22 ± 40	0.074
Energy/fatigue	38 ± 27	11 ± 13	0.003
Emotional well-being	66 ± 19	59 ± 12	0.265
Social functioning	60 ± 24	36 ± 20	0.028
Pain	65 ± 31	47 ± 23	0.141
General health	59 ± 21	36 ± 18	0.023
FACIT-F	27 ± 14	12 ± 9	0.006
BDI-II	16 ± 11	24 ± 7	0.054

Differences between the groups were analyzed using an unpaired t test

**Fig. 3:**
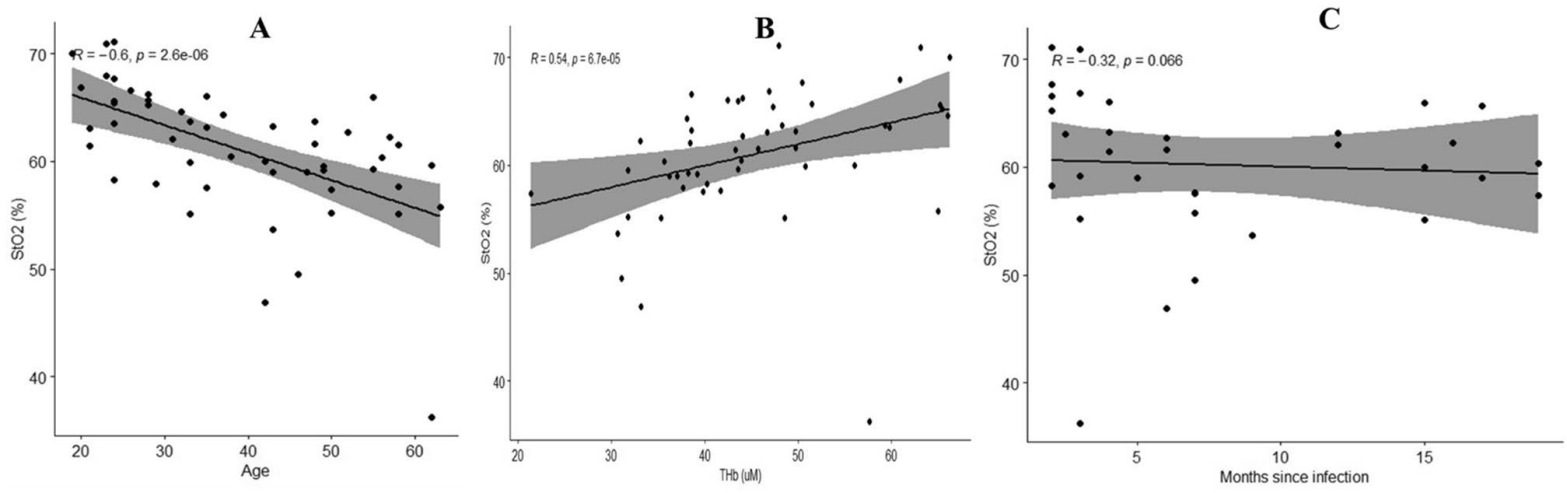
Correlation analysis between S_*t*_O_2_ (%), age (years) and total hemoglobin (THb (μM)), and months since infection, analyzed for all participants

**Fig. 4:**
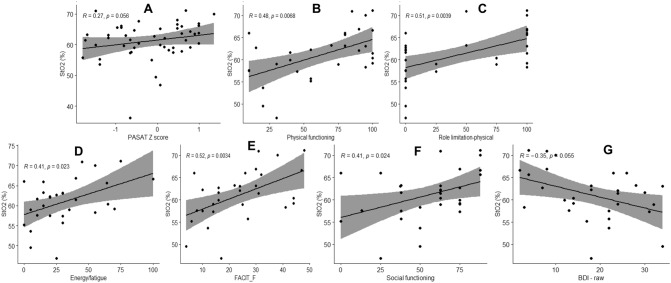
Correlation analysis between S_*t*_O_2_ (%) and paced auditory serial addition (PASAT) for healthy controls and all post-COVID-19 participants (**A**), health-related quality of life measure (physical functioning (**B**), role limitation-physical (**C**), energy/fatigue (**D**) and social functioning (**E**)), measure of fatigue (FACIT-F) (**F**) and measure of depression (BDI-II) (**G**) measured in post-COVID-19 participants only

We report the correlations between S_*t*_O_2_, as a measure of cortical microvascular oxygenation, with age, total hemoglobin (Fig. 3), and cognitive and physical functioning (Fig. 4). There was a negative relationship between age and S_*t*_O_2_ (Fig. 3A) and a positive relationship between S_*t*_O_2_ and total hemoglobin, a parameter which is related to cerebral blood volume (Fig. 3B). A correlation of months post-COVID-19 infection vs S_*t*_O_2_ was not significant (*p* < 0.066) (Fig. 3C). The slope was − 0.32 which is small and may not indicate a biologically significant change. There was a trend for a positive relationship between S_*t*_O_2_ and PASAT (Fig. 4A). We found a correlation between S_*t*_O_2_ and physical functioning (Fig. 4B), role limitation-physical (Fig. 4C), energy/fatigue (Fig. 4D), Functional Assessment of Chronic Illness Therapy-Fatigue Scale (FACIT-F measures fatigue) (Fig. 4E), and social functioning (Fig. 4F) such that reduced S_*t*_O_2_ related significantly to reduced scores. There was a negative relationship with BDI-II (measure of depression) scores (Fig. 4G). There was no relationship between systemic arterial oxygen saturation (S_*a*_O_2_) and microvascular cortical oxygenation (S_*t*_O_2_).

## Discussion

### Hypoxia

Using fdNIRS, we found that 24% of individuals, who had SARS-CoV-2 infection but were not hospitalized, had cortical microvascular hypoxia, measured at an average time frame of 7 months (range 3–15) after acute infection. Furthermore, hypoxia correlates with age, total hemoglobin, and greater symptomology like fatigue. This is despite a normal systemic oxygenation in these individuals.

A recent study in non-human primates infected with SARS-CoV-2 with mild disease presentation showed neuroinflammation and brain hypoxia [41], which is consistent with our findings. We previously proposed a “hypoxia–inflammation cycle” in multiple sclerosis [14]. It is possible that this cycle is occurring in post-COVID-19, given that both conditions involve inflammation. We believe that this hypoxia will result in reduced function and quality of life. Augustin et al*.* [42] showed that about 27.8% of SARS-CoV-2-infected individuals with mild or no disease presentation have long-term health consequences, and given the similarities between these percentages, it may be that these health consequences are related to hypoxia.

The negative relationship between S_*t*_O_2_ and age suggests that older individuals who have had the COVID-19 disease had more severe hypoxia. This is unsurprising given that it is well documented that there is an age-relate risk of developing serious complications with the COVID-19 disease [43]. Our study therefore provides further evidence supporting this.

### Inflammation and hypoxia

In post-COVID-19 condition, inflammation initially arises due to our innate immune response. Many proinflammatory cytokines are produced to eliminate viruses in the body, promoting inflammation [13]. Hypoxia-inducible factor 1 alpha (HIF-1α), the master regulator in the hypoxia response, is implicated in viral infection and innate immunity [13, 44]. HIF-1α and inflammatory cytokines have been shown to be induced in SARS-CoV-2-infected human cell lines [45]. It was proposed that upon SARS-CoV-2 infection, SARS-CoV-2 ORF3a protein induces mitochondrial reactive oxygen species to activate HIF-1α, which in turn enhances the viral infection and aggravates inflammatory responses [45]. This supports our “hypoxia–inflammation cycle” hypothesis. Furthermore, histopathological examination of brain specimens obtained from 18 patients who died 0–32 days after the onset of symptoms of COVID-19 showed hypoxia-related injury in the cerebrum and cerebellum, with loss of neurons in the cerebral cortex, hippocampus, and cerebellar Purkinje cell layer [46]. Other studies found that there was microvascular damage in the brain of individuals that died as a result of COVID-19 [47], and that there was a pronounced reduction in gray matter thickness in SARS-CoV-2-infected participants [48]. It is therefore possible that in some individuals post-COVID-19, there is SARS-CoV-2‐related microvascular damage, which may cause tissue hypoxia.

Further, a viral protease encoded by SARS-CoV-2 may cause microvascular damage and lead to neurological symptoms in COVID-19 infection [49]. This viral protease cleaves the NF-κB essential modulator (NEMO) protein, promoting neuroinflammation, brain endothelial cell death, BBB damage, and reduced CNS perfusion [49]. Evidence for microvascular damage within the frontal cortex of humans infected with SARS-CoV-2 was reported [49], the same brain region that we measured with fdNIRS in the present study. Further, patchy hypoxia was demonstrated alongside microvascular damage, endothelial cell death, and BBB damage in the brains of NEMO absent mice [49]. Using MRI, it has also been reported that in individuals with severe COVID-19 disease, there are changes in the white matter microvasculature, decrease in cortical thickness as well as reduction in cerebral blood flow, which were correlated with inflammatory biomarkers C-reactive protein, procalcitonin, and interleukin-6 [50]. It is therefore plausible that the hypoxia we report here is because of microvascular dysfunction related to these mechanisms.

We show a positive relationship between S_*t*_O_2_ and total hemoglobin, a parameter which is related to cerebral blood volume [51]. This suggests that hypoxic post-COVID-19 participants have a corresponding reduced cerebral blood volume. Mechanistically, this result could indicate that in hypoxic post-COVID-19 participants, vasoconstriction or loss of capillaries occurs, rather than vasodilation. Several studies have shown that there is microvascular damage associated with COVID-19 disease [47, 48, 52], which supports our findings.

### Light scattering and mitochondria integrity

We found differences in light scattering, where post-COVID-19 participants had a lowered scattering coefficient compared with healthy controls. The cellular nuclei and mitochondria are the most important cellular components involved in light scattering in the near-infrared region [53, 54]. Furthermore, reduced light scattering has also been suggested to relate with decreased mitochondrial density and volume [17] and loss or reduced density of brain matter [54]. We propose that scattering is a unique biomarker, which may relate to mitochondrial dysfunction and reduced density of brain matter. We did not see a detectable difference in absorption at 690 nm; however, there was a lower absorption coefficient at 824 nm. The main tissue absorbers in the near-infrared region are the oxygenated hemoglobin and deoxygenated hemoglobin in the blood. Therefore, the light absorption measured by fdNIRS mainly reflects the blood concentration and tissue oxygenation [54]. This indicates a trend to reduced blood volume in the brain of post-COVID-19 participants.

### Cognitive function, fatigue, and health-related quality of life

As frontal cortex function relates to processing speed, it is useful to note that hypoxia (S_*t*_O_2_) may impact processing speed (Fig. 4). Immune activation and inflammation in the central nervous system may be the primary driver of neuropsychological dysfunction in post-COVID-19 [8]. Given that the correlation between S_*t*_O_2_ and PASAT was weak, it is important in future studies to increase the number of study participants to see if this result can be reproduced. It is noteworthy that normoxic post-COVID-19 participants also had significantly lower scores compared with healthy controls in the visual processing speed, auditory processing speed and working memory, which suggest that deficits in these cognitive domains may be mediated by mechanisms other than hypoxia. Hypoxic participants had reduced scores for health-related quality of life, higher scores for depression, and higher levels of fatigue.

In line with previous findings, the post-COVID-19 participants reported chronic fatigue that was clinically relevant, and particularly severe in the hypoxic group [28]. Lower S_*t*_O_2_ was correlated with higher fatigue, and so it is possible the two may be mechanistically linked. Indeed, cortical hypoxia is related to fatigue and reduced exercise tolerance [55]. It may be that hypoxia, coupled with our finding of differences in light scattering which could indicate mitochondrial dysfunction, translates to fatigue. Mitochondrial dysfunction, together with hypoxia, could result in fatigue, reduced physical and social function, increased depression, and neuropsychological dysfunction, and could produce other symptoms experienced by individuals with post-COVID-19 condition.

### Strengths and limitations

There are several advantages of using fdNIRS to measure microvascular blood oxygenation as a measure of hypoxia, compared with other methods like positron emission tomography (PET) and magnetic resonance imaging (MRI). The fdNIRS system is portable, data can be collected within 3 min, and it uses low energy light to obtain HbO, and HHb concentrations, making it less invasive and allowing for frequent and repeated measurements to be made. Conversely, PET uses expensive radioactive isotopes, whereas MRI is also expensive and time consuming. fdNIRS directly measures hemoglobin concentrations, compared with MRI, which indirectly estimates the HbO saturation of large vessels by measuring the difference in susceptibility between the outside and inside of the vessel [15]. The fdNIRS system is simple to operate; therefore, measurements can be made in clinics or out in the community.

The major limitation associated with fdNIRS studies is the partial volume effect [15]. A significant portion of the NIRS signal goes through the scalp and skull before reaching the brain. Therefore, the fdNIRS signal is contaminated by the scalp and skull. If systemic oxygen levels were low, this would bias our results. The S_*a*_O_2_ values are not different in the COVID-19 group, and the arterial saturations are in a normal range. We also undertook a correlation analysis between S_*a*_O_2_ and S_*t*_O_2_ and found no correlation. These data indicate that systemic blood oxygenation is not driving our conclusions. Also due to partial volume effects, brain atrophy may influence our results, since atrophy would increase the distance from the optical fibers to the brain. We cannot rule out that brain atrophy may impact our results given that it has been shown that in individuals post-COVID-19, there is atrophy and increased tissue damage in cortical areas directly connected to the primary olfactory cortex, as well as to changes in global measures of brain and cerebrospinal fluid volume [48]. However, atrophy would result in increased S_*t*_O_2_ if, as we noted, the extracerebral tissue was normoxic. Thus, partial volume effects may be minimizing our conclusions, but would not cause the hypoxia readings.

### Conclusion

NIRS-based measures provide a unique technology that may be useful in many conditions with brain hypoxia. We have shown that 24% of people post-COVID-19 may have very low oxygen levels in the brain and that this hypoxia relates to reduced neurological function and quality of life. We have now shown that we can measure hypoxia non-invasively in individuals post-COVID-19 using fdNIRS. With this new technology, combined with neuropsychological assessment, we may be able to identify individuals at risk of hypoxia-related symptomology and so target individuals that are likely to respond to treatments that may improve oxygenation such as vasodilators, anti-clotting agents and hyperbaric oxygen therapy [56].

## Data Availability

Data will be made available upon reasonable request to qualified investigators, adhering to ethical guidelines.
